# Visual preferences for outdoor space along commercial pedestrian streets under the influence of plant characteristics

**DOI:** 10.1371/journal.pone.0264482

**Published:** 2022-03-08

**Authors:** Xulin Huang, Chenping Han, Mingkang Ma

**Affiliations:** School of Architecture and Design, China University of Mining and Technology, Xuzhou, Jiangsu, China; East China Normal University, CHINA

## Abstract

Chinese commercial pedestrian streets have developed rapidly in recent years. However, people’s preferences were not sufficiently considered and reflected in the outdoor space and landscape design. With the outdoor landscapes along commercial pedestrian streets in the region south of the Five Ridges as the research objects, this study revealed the public’s different preference evaluations of the landscapes under the reciprocal effects of street characteristics. The main results were as follows: (a) When arcade spaces were available, people prefer streets with taller trees and a lower planting density (50 plants/km or less). Conversely, they preferred streets with relatively low trees (3–6 m), a higher planting density (100–200 plants/km) and two or more vertical layers of plants. People did not like the way that plants are lined in the middle of a street. (b) When there were only one or two types of signage hanging, people preferred streets with a moderate planting density (50–100 plants/km); and there were three or more types of signage hanging, people preferred the plants with low linear density (50 plants/km or less) and that were arranged along one or two sides of the street. (c) When benches were available, people preferred streets with plants on one or both sides, fewer plant colours (one or two kind of colours) and better plant growth status. Specifically, the richer the vertical structure and the bigger number of colours were, the higher the preference score. This study provided design schemes to enhance the visual quality of landscapes by improving landscape characteristics in similar outdoor spaces.

## 1. Introduction

As important shopping and leisure sites for urban residents and tourists, modern commercial pedestrian streets not only meet people’s daily needs but also offer outdoor cultural and art stages, fitness facilities, landscape parks and other multielement living facilities. Lekagul [1] maintained that appropriate scientific research and observation on outdoor landscapes, which serve as an integral component of commercial pedestrian streets, through which the design logicality and corresponding reference value of commercial pedestrian streets as a public area can be greatly enhanced should be conducted. Therefore, the study of pedestrian street users’ visual preferences for outdoor space can improve the overall environment and walking comfort, which is of great benefit for promoting regional consumption and projecting good urban images.

Studies on the visual quality of commercial pedestrian streets have been widely conducted. Kaplan and Kaplan [2] proposed that landscape perception studies should be conducted from two dimensions, namely, spatial characteristics and plant characteristics, which are composed of specific elements of plant landscapes. Correlations exist between the spatial characteristics and the components of plant landscapes [3]. However, plant characteristics are more often than not taken as a static variable of streets in most of the previous studies. In contrast, the correlation between plant characteristics and outdoor spatial characteristics has rarely been studied. In this case, this study verifies the influence of outdoor space characteristics and plant characteristics on the evaluation of visual preference, and then reveal the effect of plant characteristics in different outdoor space on visual preference evaluation. Thus, this article aims to identify people’s preference towards pedestrian streets under the interactive influence of outdoor space characteristics and plant characteristics, for purpose of providing valuable guidance for practical design.

## 2. Literature review

### 2.1 Methods of visual preference evaluations for urban environment

Over the last few decades, many researches, conducted on interactions between the physical characteristics of a landscape and the psychological responses of those landscape recipients, has proved that streetscape photographs [4,5] or Virtual Reality [6–8] used in place of actual landscapes were considered a valid medium for such researches. Qiu [9] analyzed the relationship between subjective/objective perceptions on streetscapes and the percentage increase in sales price by using visual preference evaluations of 300 street view photos in the experiment. Ma [10] proposes a proof-of-concept analytical framework, with using millions of Tencent images to rate the complexity of the built environment, that sheds light on the connections between urban renewal and the quantification of streetscape visual traits. Based on the platform of immersive virtual reality system (IVRS), combing the isovist method and the SD method, Sun [11] analyzed the influence and effects of underground squares’ interface morphology on spatial experience. L Maftei and C Harty [12] interviewed participants about their experiences of doing design in the CAVE (Cave Automatic Virtual Environment) type of an IVR (Immersive Virtual Reality) environment. The findings of research indicate that IVRs, such as the CAVE, are enhancing existing understandings of design and driving unanticipated changes to the design.

### 2.2 Preference for outdoor space utilization in a business environment

In his study on Japanese highly popular commercial pedestrian streets, Carmelino and Hanazato [13] proposed that there are many leading factors, such as the width of the street, the walkable surface of the street, etc., that influenced the vibrancy of commercial pedestrian streets. Seresinhe, Preis and Moat [14] studied urban aesthetics with using the Places Convolutional Neural Network, and revealed the main compositions of beautiful outdoor space, such as‘Pond’, ‘Garden’ and ‘Trees’. Lu [15] and Zhang [16] also pointed out that analysis of visual preference evaluation, which using machine learning and statistical model, is an effective way to help improving the outdoor space quality.

According to Kusumarini, de Yong, and Thamrin [17], good signage placement could effectively influence how well people recognize the commercial information conveyed by the signage, which would help them locate the commodities they wanted to buy and in turn improve the comfort level of the spatial experience. As for the setting of signage in a commercial environment, Motamedi et al. [18] observed that signage design should give priority to its significance, readability, and legibility. Xie [19] revealed that the hanging modes of shop signage could be divided into different types, such as horizontal, wall hanging, vertical and external. The diversity of signage also contributes to improving the significance of signage.

Along commercial pedestrian streets in South China, there are many single-span arcades that serve as the transition between streets and buildings. According to Peng and Yang [20], the arcade space, which is called “Qilou” in Chinese, is the architectural form widely seen along the streets in the region south of the Five Ridges. It embodies a strong sense of civilian consciousness. Geist [21] maintained that the arcade space could effectively protect pedestrians against the influence of traffic and weather and promote the development of product exchange markets. The arcade space on the ground floor can enhance the permeability of street-facing façades and enrich the dimensions of pedestrians’ spatial experience, which in turn attract more people to visit. Hassan, Moustafa, and El-Fiki [22] also stated that the benches, awnings and product display posters offered by street-side shops could enhance the spatial permeability of pedestrian streets. In this sense, the setting of benches should be encouraged to promote the vibrancy of commercial pedestrian streets. However, the seat setting should be regulated so as to guarantee comfortable walking space for pedestrians [23]. Furthermore, the streets are usually divided into street sequences by various facilities and border lines, forming different visual perceptions [5,24,25].

Based on the abovementioned related literature, four outdoor spatial characteristics of pedestrian streets were set as influencing factors to study the visual preferences for commercial pedestrian streets. The four outdoor spatial characteristics were as follows: arcade space, number of street sequences, benches and number of signage hanging types. This research aimed to check whether the four selected characteristics exerted any influence on visual preferences.

### 2.3 Preference for plant characteristics in business environment

For modern urban residents, casual strolling has become an important form of relaxation and entertainment. The quality of outdoor plant landscapes is usually regarded as one of the five attributes of pedestrian streets that influence strolling activities [26,27]. Through effective management and design, the outdoor plant landscapes in commercial pedestrian streets can attract more consumers [28,29], greatly reduce the environmental pressure imposed on pedestrians by urban corridors and enhance pedestrians’ comfort level in the process of strolling [30–32].

In addition, landscape evaluation may differ when different types of plants are involved, such as trees with different heights [24,33,34] or different arrangements, which in turn exert spatial separation and guide feelings [25,35,36].

Accordingly, to explore the quantitative influence of different plant characteristics on visual preferences, this study summarized seven common plant characteristics including vertical structure, arrangement, growth status, number of colours, height of trees and linear density of planting. The seven variables were obtained through the analysis of landscape characteristics observed by other scholars [37–40]. This study may provide a reference for outdoor plant landscape design and management along commercial pedestrian streets.

Therefore, based on a case study of commercial pedestrian streets in typical cities in southern China, this study investigated the evaluation of customers’ visual preferences for outdoor spaces and plant landscape design. The objectives of this study are as follows:

1. To investigate the factors of public outdoor spaces that influence the visual preferences for pedestrian streets.
2. To investigate the factors of outdoor landscape design that influence the visual preferences for pedestrian streets.
3. To evaluate the effect of plant characteristic in different outdoor space on visual preference evaluation.

## 3. Methods

### 3.1 Study area and sites

This study selected six cities from two provinces, namely, Nanning, Wuzhou and Liuzhou in Guangxi Province and Guangzhou, Shenzhen and Huizhou in Guangdong Province. Located in the region south of the Five Ridges, all six selected cities are highly representative of tropical and subtropical climates with a long hot and humid summer. In this case, there is a comparatively strict requirement upon the degree of coverage of shelters in outdoor public space. In addition, the hot and humid climate makes very durable plant landscapes possible. Therefore, it is of typical significance to select the outdoor landscape setting along the commercial pedestrian streets in these cities as research objects [41,42].

### 3.2 The shooting of the photos used in the study

The photos used in this study were taken by the author in the abovementioned cities in September 2019. All photos were taken at the common visual height (approximately 165 cm vertical from the ground) during the period from 10 a.m. to 4 p.m. in a sunny or cloudless morning or afternoon to guarantee the lighting conditions. In total, 210 photos were shot and grouped in accordance with the similarity of the stimulating factors. Finally, 25 photos with good photographic quality were selected by ten professional architects and landscape designers for the questionnaire survey [28,43], and the criteria were as follows: good photo quality, sufficient outdoor space factor and plant factor. The method of using photos rather than actual views to conduct visual preference evaluation has been widely used in existing studies [44–46].

### 3.3 The judgement of outdoor spatial and the plant landscape characteristic classification

The value of variables denoting four different outdoor spatial characteristics and seven different plant characteristics in Tables 1 & 2, selected according to above-mentioned literature, were determined by 10 professionals [27,28,45,47,48]. Three architects, five landscape designers, and two forestry experts were invited to evaluate and judge the value of each variable listed in Tables 1 and 2, respectively. The 25 photos plus one e-questionnaire were emailed to the ten professionals who were asked to complete the questionnaire independently.

**Table 1:** Measurement scale of outdoor spatial characteristics.

Outdoor spatial characteristics of commercial pedestrian streets	The value of variables denoting different characteristics
Arcade space	available = 1 and unavailable = 2
Number of street sequences	one sequence = 1, two sequences = 2, and three or more sequences = 3
Number of signage hanging types	one type = 1, two types = 2, and three or more types = 3
Benches	available = 1 and unavailable = 2

**Table 2:** Measurement scale of plant characteristics.

Plant characteristics	The value of variables denoting different characteristics
Vertical structure of plants	One layer = 1, two layers = 2, and three or more layers = 3
Arrangement of plants	Along one side of the street = 1, along two sides = 2, and in the middle of the street = 3
Height of trees	3–6 m = 1, 6–12 m = 2, and 12 m or higher = 3
Growth status of plants	poor = 1, intermediate = 2, and good = 3
Number of colours	one kind of colour = 1, two kinds of colour = 2, and three or more kinds of colour = 3
Linear density of planting	low(50 plants/km or less) = 1, intermediate(50–100 plants/km) = 2, and high(100–200 plants/km) = 3

Technical terms were used when the characteristics (as listed in Tables 1 & 2) were classified so that the professionals could evaluate the characteristics more carefully and accurately [40]. When they completed the questionnaire, the professionals returned it to the author before the deadline. The value of variables for each photo is determined by the feedback judgement of ten professionals.

### 3.4 Questionnaire survey

Photo stimulation was used as the major research method of the questionnaire survey in this study. The photo stimulation method is widely used as an effective way to represent an actual environment with photos [39,49,50]. All 25 photos were printed in full colour on A4 paper. The survey participants consisted of pedestrians residing in the Nanning who were Chinese and above 18 years old. Nanning, which is located in the middle of Guangxi Province, is of a typical warm, monsoon-influenced humid subtropical climate, with an annual mean temperature of 21.83°C (71.3°F). The climatic characteristics and economic conditions of Nanning are representative in Guangdong and Guangxi area, which can effectively reflect the pedestrian’s willingness and the visual preference of the landscape in this area. The participant recruitment method in this study was that every person who walked out at one identified area near the main entrance of the pedestrian streets had the same probability of participating in this study. In doing so, one participant was approached every 10 min at a specific location near the main entrance. However, if any of the participants refused to answer the survey questionnaire, the next participant who walked out of the same area was selected. Ten minute intervals were suitable for this study. Participants took an average of 5 to 7 minutes to answer the questionnaire, with the longest time being 9 minutes.

During the survey, the participants could browse the printed photos at will, review the score of a photo, or even rescore a photo. Scene preference was scored by five grades (1–5): 1 denotes the least attractive scene of pedestrian streets whereas 5 denotes the most attractive scene. No specific permits were required for these activities, because these studies did not involve protected plant species or area of land. All these surveys have obtained participants’ oral consent.

The first survey was conducted in July 2019, and another survey was conducted in September 2019, from which 392 questionnaires were collected in total. The four pedestrian streets with high traffic and popularity [51,52] in Nanning, Xingning Road, Zhongshan Road, Chaoyang Road and Gonghe Road, were identified as the survey locations. Since the preference scores obtained from the two rounds of survey were similar to each other (as the one-way ANOVA indicated; F = 0.532, p = 0.479), all the questionnaires collected (208+184 = 392) could be used for further analysis.

Suhardi [53] proposed that when the error of the experimental sample size is less than 5%, the sample can represent a large number of people of a certain type. A formula used to test the sampling error could increase the confidence level of the experimental sample while simultaneously reducing the nonsampling error. For this study, the proposed sample size was 392 to reduce both sampling and nonsampling errors. Therefore, the sampling error based on the above formula is: sampling error = square root of [(p) (1-p)/proposed sample size], e = √ [(p) × (1−p)/sample size] = √ [(0.5) × (0.5)/392 = 2.52%.

### 3.5 Ethics statement

The topic is not ethically sensitive and was carried out in accordance with national and institutional legal and ethical requirements. Data were collected completely anonymously (i.e. no possibility to reidentify whatsoever) and therefore this work falls outside the scope of GDPR 2016 and MDSM (Measures for Data Security Management) for China.

The project follows institutional guidelines and was discussed with the internal ethics reference person who indicated that there was no need for ethical approval when surveys are not directly health related. In China, there is no legal requirement for ethical approval of such a survey where no sensitive issues are explored and no privacy is involved, and there are no IRB mechanisms in place for this type of work. Sensitive data or research involving human subject undergo ethical approval through ethical research committees based in hospitals that do not assess this type of projects.

Also, ethical concerns were assessed internally: participation was on a voluntary basis and all participants were informed that the survey was anonymous, that all data would be only used for research and evaluated anonymously. To secure privacy, all data was collected and analysed anonymously with no collection of identifiers/codes.

### 3.6 Data analysis method

The mean scores of the photos were analysed with SPSS 22.0. To begin with, the one-way ANOVA analysis was conducted to check whether the four outdoor spatial characteristics exerted any impact on preference evaluation. Then correlation analysis was conducted to study the relationship among the four outdoor spatial characteristics. On this basis, stepwise multiple linear regression analysis was conducted to analyse the qualitative relationships between the four outdoor spatial characteristics and preference evaluation. Similarly, the quantitative relationships between plant characteristics and preference evaluation under the influence of different outdoor spatial characteristics was explored in the same way. These analytical methods were widely employed in previous similar studies [13,44,46,54].

## 4. Results

### 4.1 The overall evaluation of the photos

First, the preference evaluation scores of the 25 photos from participants in pedestrian streets were processed to analyse the interclass reliability. The results indicated that the Cronbach’s alpha was 0.796, confirming that the data collected shared relatively high interclass reliability.

**Fig 1:**
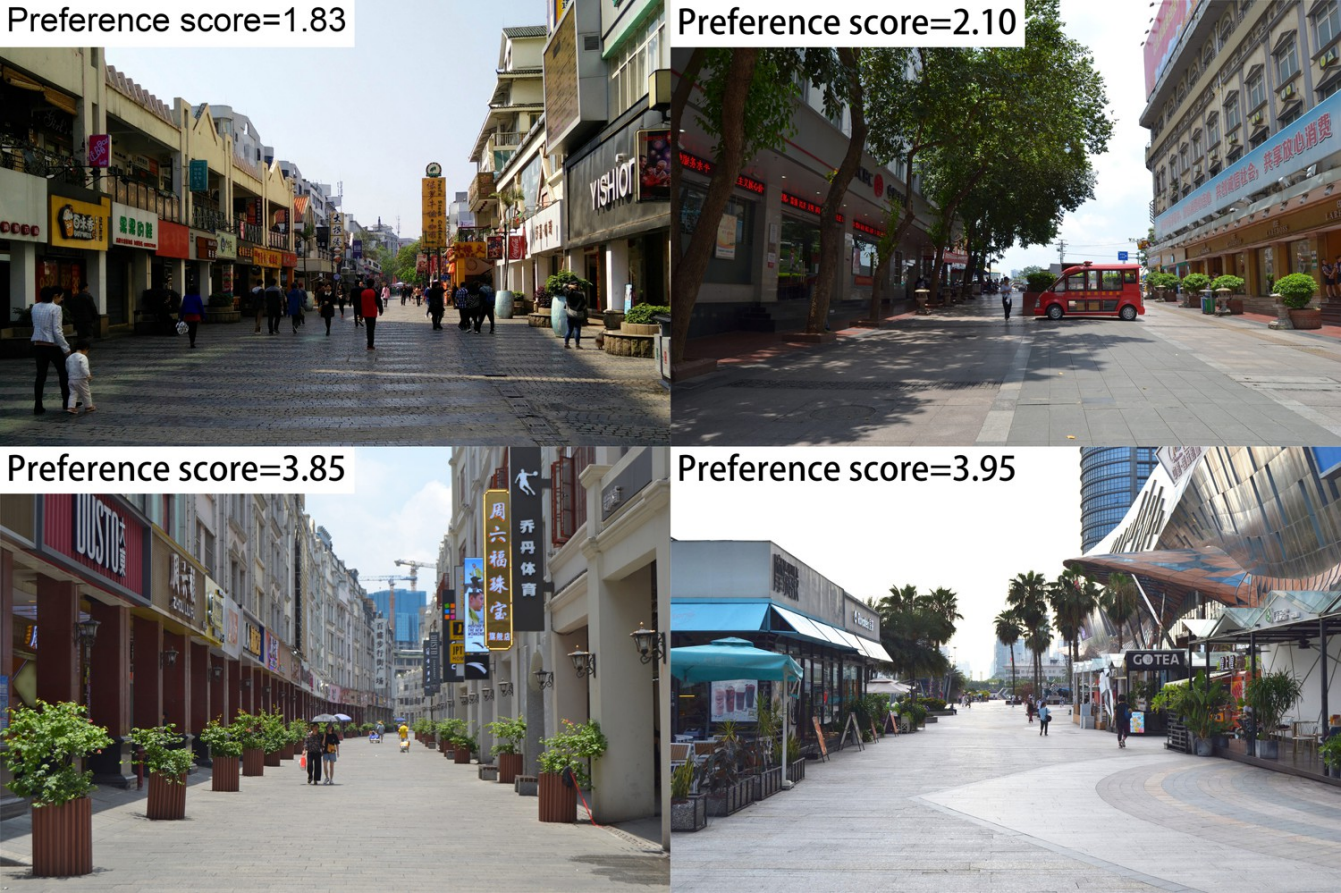
The photographs with the lowest preference scores and highest preference scores.

Then, the mean evaluation scores of all 25 photos given by the pedestrian participants were calculated. The mean evaluation score was 2.94, the highest score was 3.95 and the lowest score was 1.83. Fig 1 show the top two photos with the lowest scores and highest scores, respectively.

### 4.2 Outdoor spatial characteristics of pedestrian streets and landscape preference evaluation

The one-way ANOVA revealed that three of the four outdoor spatial characteristics could produce significant differences, namely, arcade space (F = -3.434, p<0.001), benches (F = -3.616, p = 0.001), and number of signage hanging types (F = 8.624, p = 0.003). As for the number of street sequences, since the Levene homogeneity test of variance showed that the variance of the grouped data was heterogeneous, nonparametric tests were conducted in this case. The test results indicated that the number of street sequences did not produce any significant difference in preference scores (F = 2, p = 0.563).

Similarly, Spearman correlation analysis (suitable for analysis between categorical variables and continuous variables) showed that preference evaluation was significantly correlated with arcade space (negative correlation), the number of signage hanging types (positive correlation), and benches (negative correlation), but there was no significant correlation detected between preference evaluation and the number of street sequences (as shown in Table 3).

**Table 3:** Correlations between landscape preferences and pedestrian street characteristics.

		Preference evaluation score	Arcade space	Number of street sequences	Number of signage hanging types
Arcade space	coefficient	-0.362*			
	significance	0.028			
Number of street sequences	coefficients	0.021	0.236		
	significance	0.693	0.071		
Number of signage hanging types	coefficients	0.487**	0.051	-0.216	
	significance	0.009	0.634	0.198	
Benches	coefficients	-0.851**	0.124	-0.175	-0.194
	significance	0.001	0.258	0.316	0.067

* Correlation is significant at the 0.05 level.

** Correlation is significant at the 0.01 level.

Through correlation analysis, arcade space, number of signage hanging types, and benches could be determined to be the independent variables of the experiment; the mean preference score of the 25 photos was set as the dependent variable. On this basis, stepwise multiple linear regression analysis was conducted to describe the above-mentioned significant correlations. The results of regression analysis revealed that arcade space, number of signage hanging types, and pedestrian seats did exert significant influence on preference evaluation (Table 4).

**Table 4:** Significant predictors (outdoor space characteristics) for 25 photos’ mean preference score of each participant emerging from the stepwise multiple linear regression analysis.

Model		Unstandardized Coefficients		Standardized Coefficients	t	Sig.	Collinearity Statistics	
		B	Std. Error	Beta			Tolerance	VIF
	(Constant)	4.121	0.350		11.788	0.000		
	arcade space	-0.565	0.223	-0.389	-2.528	0.004	0.880	1.136
	number of signage hanging types	0.407	0.155	0.624	3.672	0.013	0.796	1.342
	benches	-0.478	0.197	-0.519	-3.076	0.008	0.832	1.216

a. Dependent Variable: Mean preference score.

To verify the model correctness, the Kolmogorov-Smirnov test was conducted to analyse the normality of the residuals, the variance, and multicollinearity. The results show that the residuals were distributed normally (Kolmogorov-Smirnov Z = 0.642, p = 0.513 > 0.05). Arriaza [4] and Lin L [44] proposed that when the value of the tolerance was smaller than 0.2 or the variance inflation factor (VIF) was larger than 10, there would be collinearity in the experimental model. As for the model established in this study, there was no multicollinearity because its value of tolerance was 0.880 (larger than 0.2) and its VIF was 1.136 (smaller than 10). Accordingly, the model was reliable.

### 4.3 Preference evaluation under the influence of outdoor spatial factors and plant characteristics

The interrelationship between plant characteristics and outdoor spatial characteristics was also explored. As introduced above, the availability or unavailability of arcade space and benches and the different number of signage hanging types were valued differently. In this case, the mean preference scores of the corresponding photos were set as the dependent variable, and the seven plant characteristics were set as the independent variables to conduct stepwise multiple linear regression analysis, although the number of signage hanging types was not significantly correlated with plant characteristics. The results of the regression analysis indicated that the reliable predictors obtained were not consistent in different cases (Table 5).

**Table 5:** Analysis of the relationship between plant characteristics and outdoor spatial characteristics.

Dependent	Independent	Unstandardized Beta	Standardized Beta			Collinearity Statistics	
				t	Sig.	Tolerance	VIF
Preference evaluation (with arcade space) (adjustment: R^2^ = 0.268, n = 15)	Height of trees	0.542	0.323	1.363	0.017	0.756	1.347
	Linear density of planting	-0.413	-0.285	-1.417	0.037	0.629	1.472
Preference evaluation (without arcade space) (adjustment: R^2^ = 0.247, n = 10)	Height of trees	-0.691	-0.527	-1.607	0.014	0.876	1.141
	Vertical structure of plants	0.838	0.653	1.952	0.002	0.898	1.121
	Arrangement of plants	-0.429	-0.310	-1.246	0.042	0.981	1.038
	Linear density of planting	0.597	0.481	1.317	0.012	0.673	1.376
Preference evaluation (with only one type of signage hanging) (adjustment: R^2^ = 0.312, n = 4)	Linear density of planting	-0.719	-0.585	-1.712	0.006	0.882	1.132
Preference evaluation (with two types of signage hanging) (adjustment: R^2^ = 0.487, n = 9)	Linear density of planting	-0.846	-0.583	-1.952	0.003	0.852	1.163
Preference evaluation (with three or more types of signage hanging) (adjustment: R^2^ = 0.361, n = 12)	Arrangement of plants	-0.429	-0.261	-1.451	0.024	0.812	1.421
	Linear density of planting	-0.856	-0.523	-1.746	0.005	0.842	1.276
Preference evaluation (with benches) (adjustment: R^2^ = 0.603, n = 12)	Arrangement of plants	-0.617	-0.485	-2.374	0.013	0.841	1.121
	Number of colours	-0.319	-0.291	-2.105	0.043	0.823	1.142
	Growth status of plant	0.561	0.417	2.413	0.024	0.792	1.267
Preference evaluation (without benches) (adjustment: R^2^ = 0.628, n = 13)	Vertical structure of plants	0.518	0.593	1.852	0.011	0.719	1.390
	Number of colours	0.592	0.637	1.246	0.027	0.683	1.465

#### (1) The reciprocal effect between preference evaluation under the influence of arcade space and plant factors

**Fig 2:**
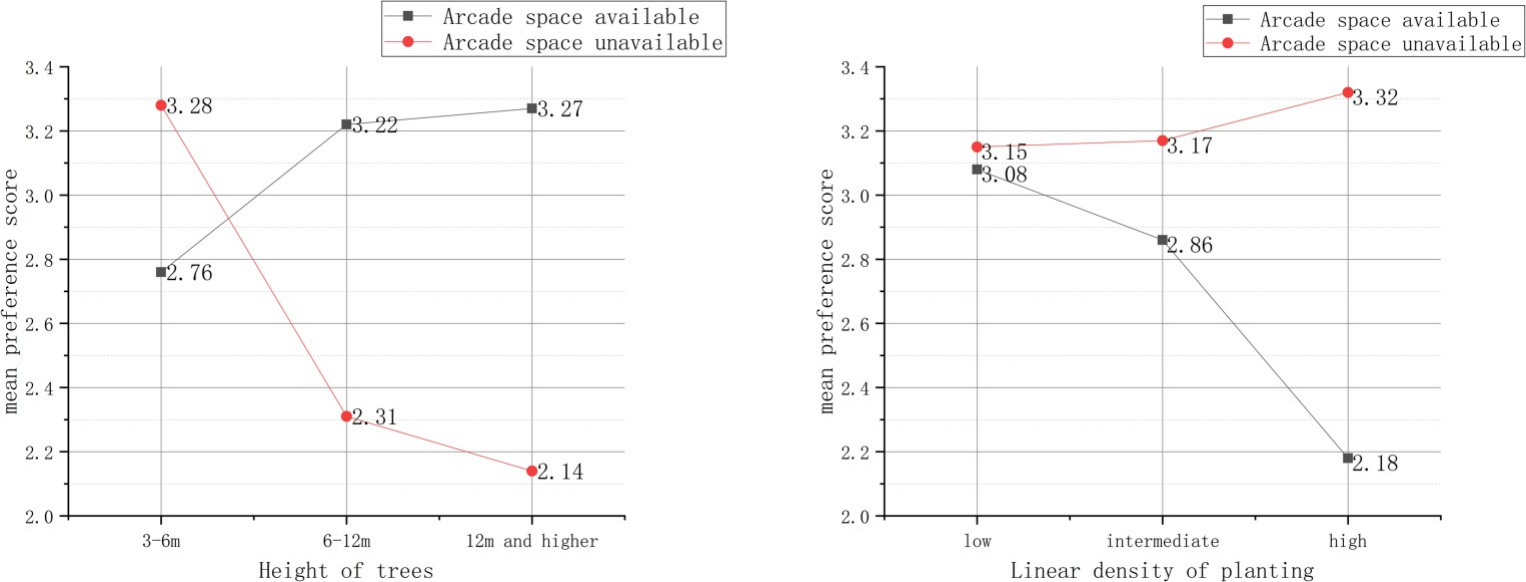
Height of trees and linear density of planting.

The mean preference score indicated that when arcade space was available along pedestrian streets, the preference score significantly increased as the height of trees increased; and when the linear density of planting was high, the preference score showed a remarkable decreasing trend compared with the other two group scores. With unavailable arcade space and the height of trees within the range of 3–6 m, the preference score showed a remarkable increasing trend compared with the other two group scores. The higher the linear density of planting was, the higher the preference score (Fig 2).

**Fig 3:**
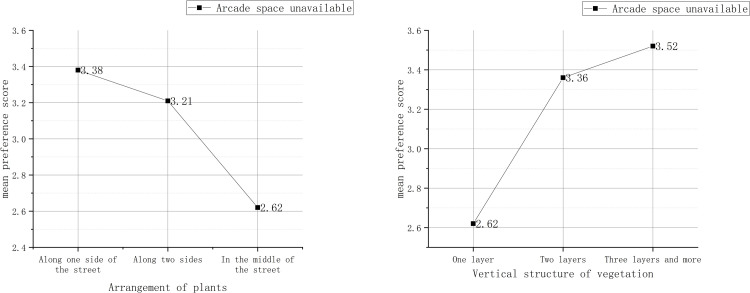
Arrangement of plants and vertical structure of plants.

In addition, when arcade space was unavailable, the vertical structure of plants and the arrangement of plants attracted more concern. The diverse vertical structure could effectively increase the preference score. When the plants were arranged along one or two sides of a street, the preference score was also higher than that when the plants were placed in the middle of the street (Fig 3).

#### (2) The reciprocal effect between preference evaluation under the influence of the number of signage hanging types and plant factors

**Fig 4:**
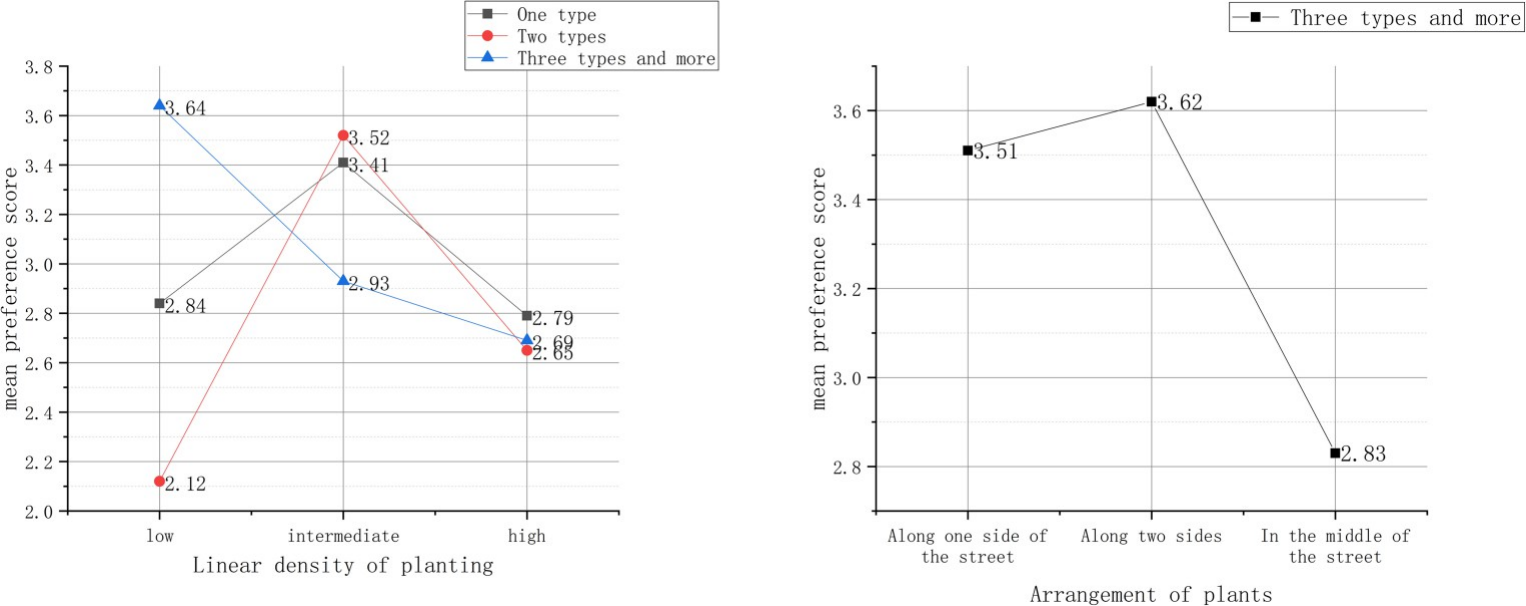
Linear density of planting and arrangement of plant.

Furthermore, this study discovered that the linear density of planting at an intermediate level when there were only one or two types of signage hanging, the preference score showed a remarkable increasing trend compared with the other two group scores. When there were three or more types of signage hanging, people preferred the plants with low linear density and that were arranged along one or two sides of the street (Fig 4).

#### (3) The reciprocal effect between preference evaluation under the influence of benches and plant factors

**Fig 5:**
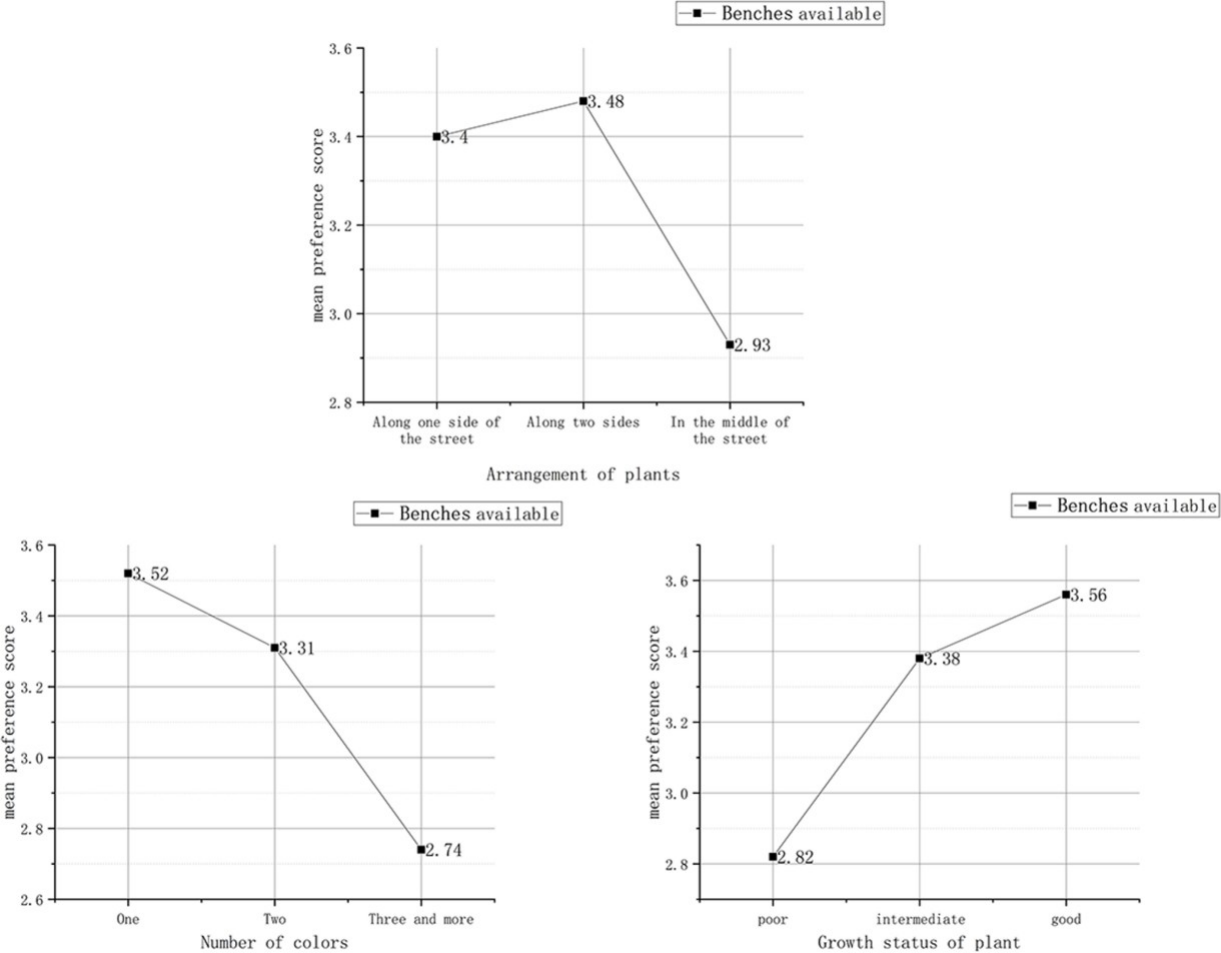
Preference evaluation under the influence of benches and plant factors Ⅰ.

In addition, this study discovered that when there were benches on the street, the crowd paid more attention to the “arrangement of plants”, the “colour”, and the “growth status of plants” (as shown in Table 5); and when plants were arranged along one or two sides of street, the preference score was significantly higher than the third arrangement. As for the number of colours, the smaller the number of colours were, the higher the preference score was. Conversely, the better the plants grew, the higher the preference score (Fig 5).

**Fig 6:**
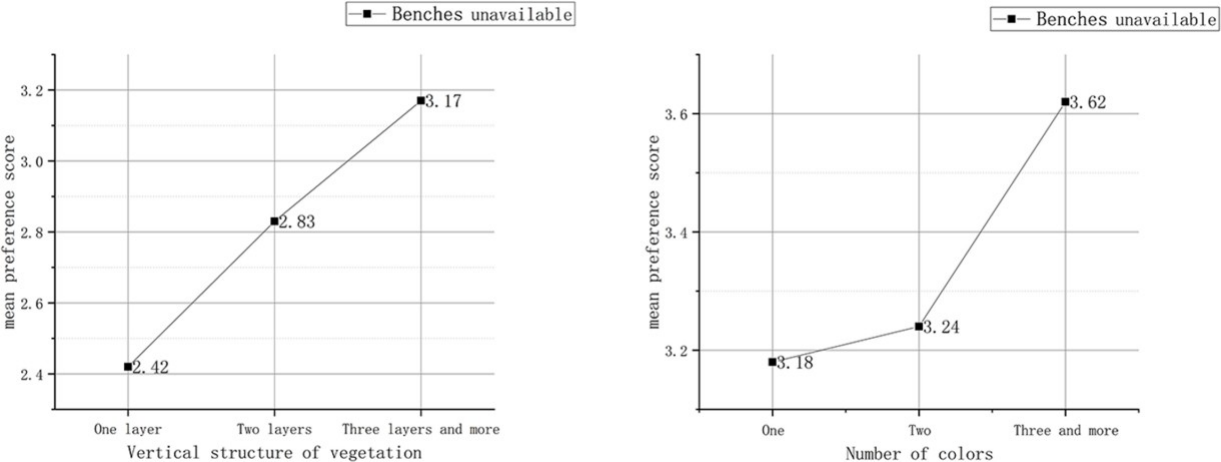
Preference evaluation under the influence of benches and plant factors Ⅱ.

As for the streets without benches, the crowd paid more attention to the vertical structure and number of colours of plants (Table 5). Specifically, the richer the vertical structure and the bigger the number of colours were, the higher the preference score (Fig 6).

## 5. Discussion

Based on the results obtained, this study verified that arcade space, the number of signage hanging types and benches exerted significant influences on visual preference evaluation (Tables 3 & 4).

The data analysis (Table 4) indicated that pedestrian streets with arcade space scored relatively higher than those without arcade space. This was in line with the research results of Peng and Yang [20] who studied the arcade space in Southeast Asia. According to Kang, Fukahori, and Kubota [55], streets with large amounts of semiprivate space, such as arcade space, would induce more potential actions and be more effective for designing attractive roadside spaces by providing pedestrians with an opportunity to interact with roadside spaces and facilities.

As for the influence of the number of signage hanging types on visual preference, Table 4 demonstrates that diverse types of signage hanging can promote the visual preference evaluation of pedestrian streets. According to Motamedi et al. [18], signage design should stress the significance, readability and legibility of signage. Therefore, the diversity of signage types should be correlated with the significance of signage. Diverse signage designs and hanging types can attract pedestrians’ attention to commercial space. Compared with the single hanging type, diverse signage hanging types can reduce pedestrians’ visual fatigue and promote the significance of signage while enriching their visual experiences.

As for the influence of benches on visual preferences, the data in Table 4 verified Mahmoudi, Ahmad, and Abbasi [56]: as long as benches, shade, and facilities for the disabled on the pedestrian streets are arranged reasonably and appropriately, they can effectively improve the comfort level of outdoor space.

### 5.1 plant characteristics and arcade space

As revealed above, the preference scores of pedestrian streets with or without arcade space would vary differently as the height of trees and linear density of planting change. Through observing and comparing the photos selected for the experiment, the author summarizes the reasons as follows: (1) In urban areas of South China where the weather is relatively hot and humid, trees three to six metres high can provide shade for pedestrians beneath them. (2) Trees three to six metres high can effectively block business signs on façades that are disharmonious with the surroundings and out of order. The plant landscapes reshape the rhythm and rhyme of the façade of pedestrian streets. This is consistent with the research of Shi, Gou, and Chen [57] and Elsadek et al. [58] who observed that the enclosure formed by plants along the streets would influence pedestrians’ mental feelings.

Furthermore, as shown in Fig 2, a higher linear density of planting can further strengthen this effect when there was no shady space such as arcade space, which verifies the positive correlation between the linear density and preference score. In contrast, when there was outdoor shady space such as arcade space on a street, high trees and a relatively low density of planting can function as positive elements to enrich the façade of the pedestrian street interface without disturbing pedestrians’ mental feelings regarding the key attributes of pedestrian streets such as the business atmosphere and architectural style. This is in line with the research of Othmana et al. [46] who maintained that shade trees should offer pedestrians good visual feelings and environmental anticipation.

As shown in Fig 3, when there is no arcade space, plants that are placed in multiple layers along one side or two sides of the street can form a corridor-like outdoor space. In this case, pedestrians can perceive and accept the overall design more easily. Moreover, multilayer plant landscapes are definitely more sensuously stimulating to pedestrians than single-layer landscapes. The interface is more adaptable for remoulding, thus being more likely to be reorganized into a wholeness by pedestrians.

### 5.2 Plant characteristics and number of signage hanging types

Table 5 shows the effect of plant characteristics with different signage hanging types on preference evaluations. This can be interpreted by the theory proposed by Motamedi et al. [18] that signage design should be significant, readable, and legible. When the signage types are relatively simple and monotonous, taller trees will not block orderly arranged business signs, thus guaranteeing the viewability of signs and effectively promoting the recognizability of commercial pedestrian streets [59]. As shown in Fig 4, an intermediate density of planting can not only relieve the visual fatigue caused by a monotonous interface but also guarantee that the signs are not blocked too much. In this case, the facade interface could be maximally unified and harmonious. When the signage types are relatively plenty, a relatively lower density of planting is more advisable since this arrangement will block the signs to a lesser extent and thus guarantee the unity and cleanliness of the facade, which results in higher preference scores.

### 5.3 Visual preference difference caused by plant arrangement with or without benches

As the photos show, benches are mostly arranged along the two sides of a street where the plants can provide excellent shade over the seats. Table 5 and Fig 5 can be interpreted with the laws of Gestalt psychology [60]. In the photos, the benches partially block the plants, thus projecting a visual front-back relationship. According to the principle of similarity, plants that are the same or similar to the arrangement of benches and a relatively small number of plant colours would work together to form similar patterns and backgrounds. In this case, it is difficult for pedestrians to perceive every individual attribute. Instead, all the attributes and elements form an overall pattern that is accepted by the crowd. Plants with a good growth status, complete appearance status and harmonious colour can be assimilated into the outdoor space.

If there are no benches, the plants will not be blocked at all. In this case, they can be presented fully in the photos and become the main source of visual stimulation for the crowd. This also verifies the importance of rich facade elements to good preference evaluation, which was proposed by [22]. The diverse landscape layers and number of colours enrich the façade layers and sensuous experience, thus enhancing the preference evaluation score (as shown in Fig 6).

### 5.4 Limitations and future research direction

The selection of plant species is a limitation of this study. For example, when selecting trees or shrubs for plant landscapes, there are actually many choices in the subtropical region south of the Five Ridges. According to Bircher and Bircher [61], Palmae and Sterculiaceae are widely used as shade trees, and Rubiaceae and Magnoliaceae are quite popular shrubs for plant landscapes. However, this study only used the shrubs and trees in the photos as landscape parameters to conduct the factor intervention. Although this method has been widely used by other researchers [5,24,46,62], more plant species should be selected in future research to verify their influence on visual preference evaluation.

In conclusion, since the landscape evaluation of pedestrian streets is subjective, this study endeavoured to establish the relation between the plant landscape and other landscape attributes as objectively as possible and provide reliable guidance for plant landscape design for pedestrian streets with different conditions.

## 6. Conclusion

In summary, this study summarized the importance of arcade space, the number of signage hanging types, and benches to visual preference evaluation using the qualitative outdoor spatial characteristics of pedestrian streets and pedestrians’ visual preference evaluation. It is concluded that in the subtropical region south of the Five Ridges, priority should be given to the creation of outdoor shade space, such as arcade space and shade trees. Besides, appropriate benches and outdoor plant landscapes would be of great help to create well-organized and highly permeable commercial space.

In addition, as for outdoor spaces with different characteristics, this study proposed specific methods for selecting the appropriate plant characteristics so as to maximize the visual enhancement. Specifically, for the pedestrian streets with arcade spaces, visual preference evaluation can be improved by selecting relatively higher trees and a lower density of planting. As for pedestrian streets without arcade space, the height of trees should be relatively lower; furthermore, multilayer and high-density shrubs can be used to remould the facades along one or two sides of pedestrian streets.

This study also offered different specific suggestions for selecting plant characteristics for pedestrian streets with or without benches. Similarly, suggestions were also offered for streets with different number of signage hanging types.

As with the experiment conducted by Santosa, Ernawati, and Wulandari [63] who used the public’s participation to improve the visual quality of streets, community participation can have a positive effect on pedestrian streets by protecting and activating pedestrian streets with low activity. For future research, it is advisable to adopt some methods that involve more public participation, such as questionnaire surveys and virtual scene restoration, to reveal the internal relationships between preference evaluation and the attributes of architectural space and provide valuable guidance for practical design.

## Supporting information

S1 Raw images (PDF)
