# Supplementary material for: Visual preferences for outdoor space along commercial pedestrian streets under the influence of plant characteristics
Source: PLoS One. 2022 Mar 8;17(3):e0264482. doi: 10.1371/journal.pone.0264482 (PMC8903252; doi:10.1371/journal.pone.0264482)
Supplement: S1 Raw images — (PDF) [file pone.0264482.s001.pdf]

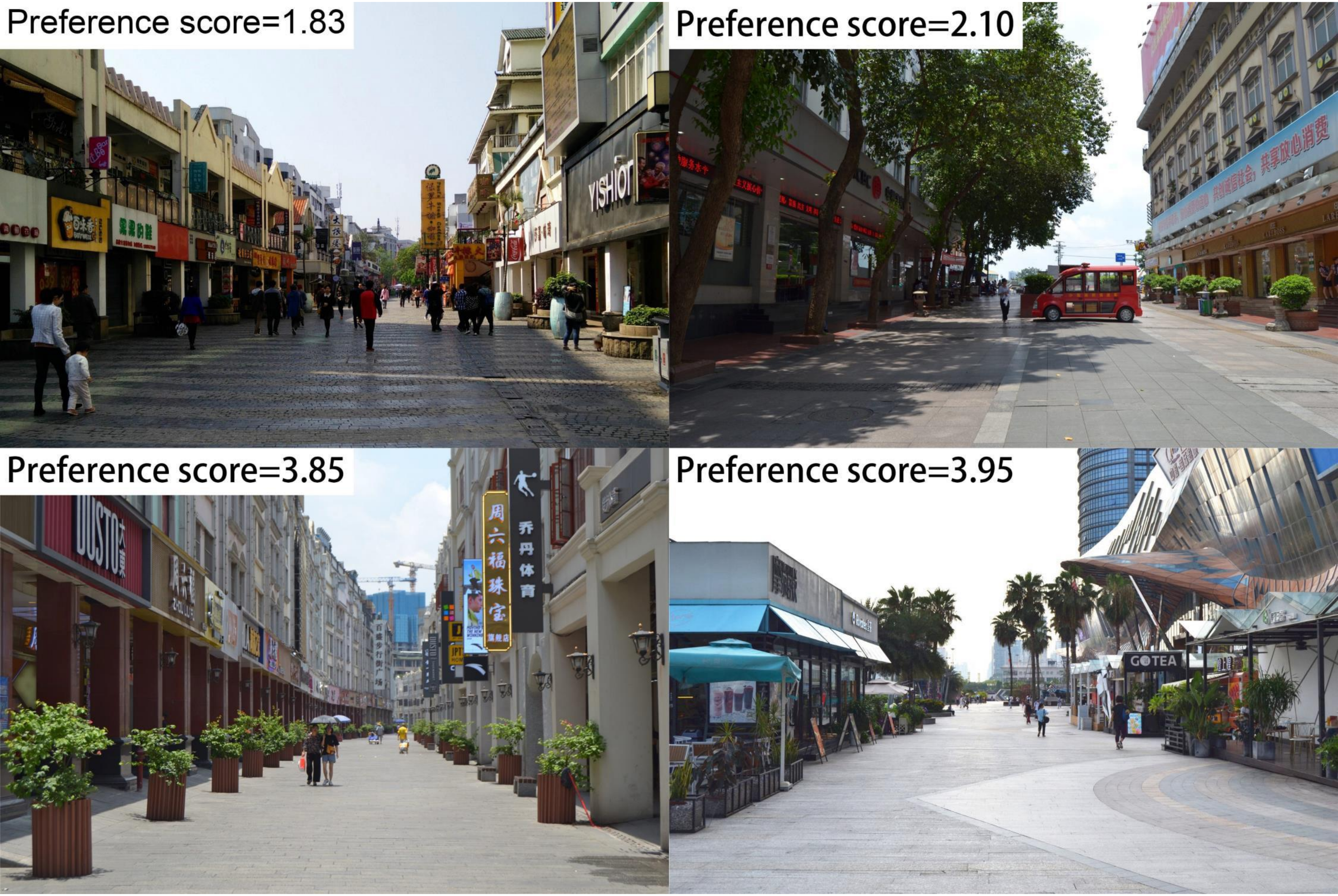

Fig 1 The photographs with the lowest preference scores and the highest preference scores

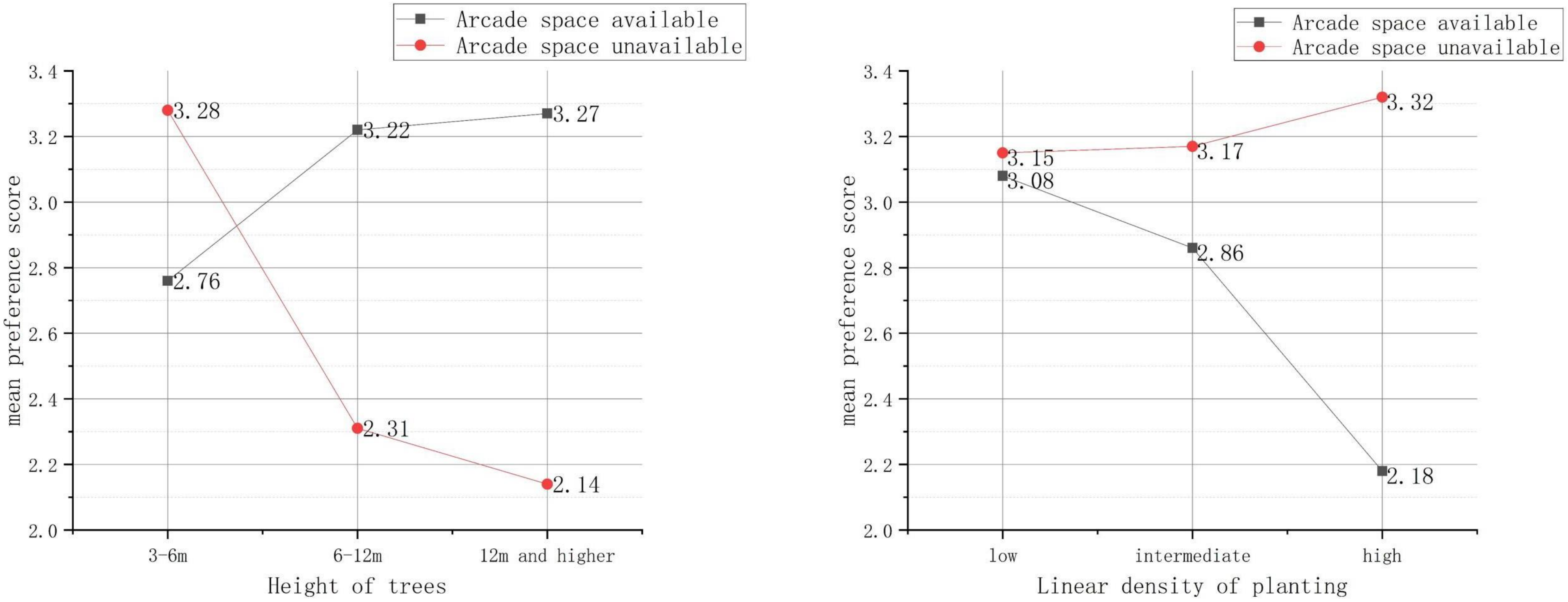

Fig 2 Height of trees and linear density of planting

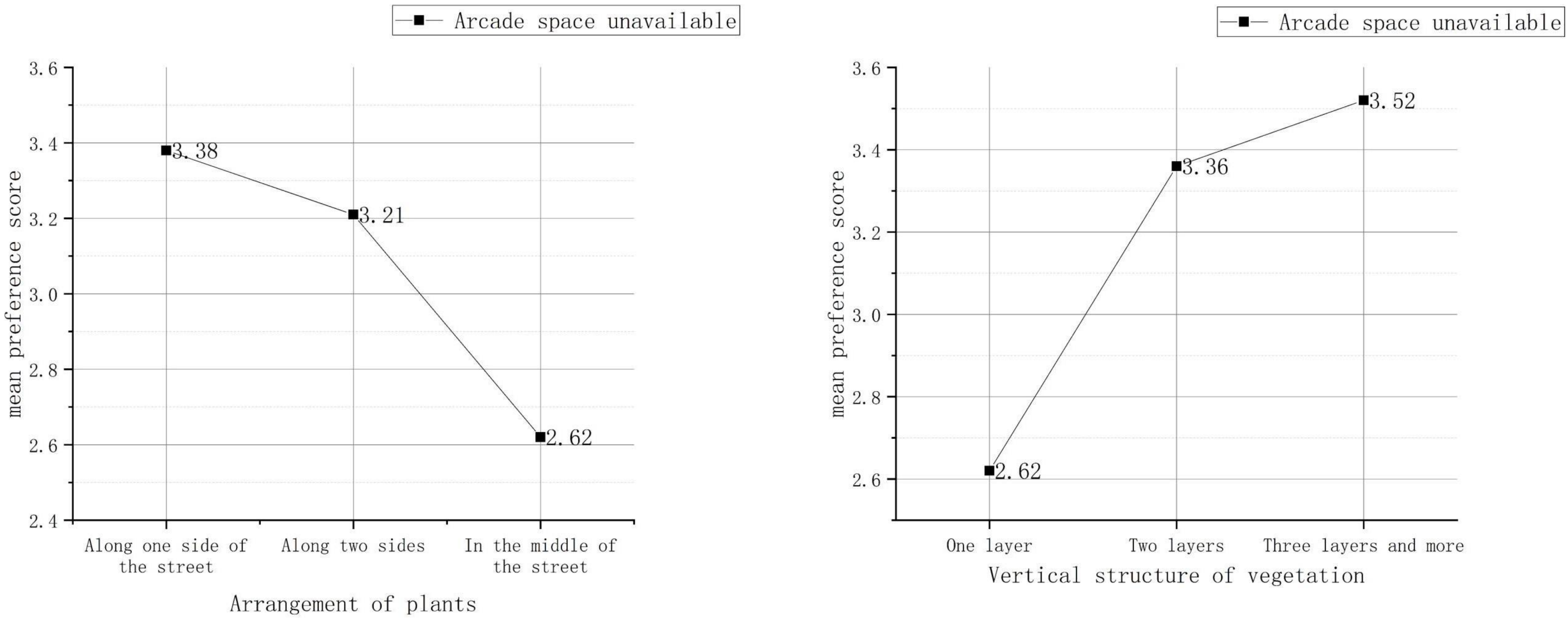

Fig 3 Arrangement of plants and vertical structure of plants

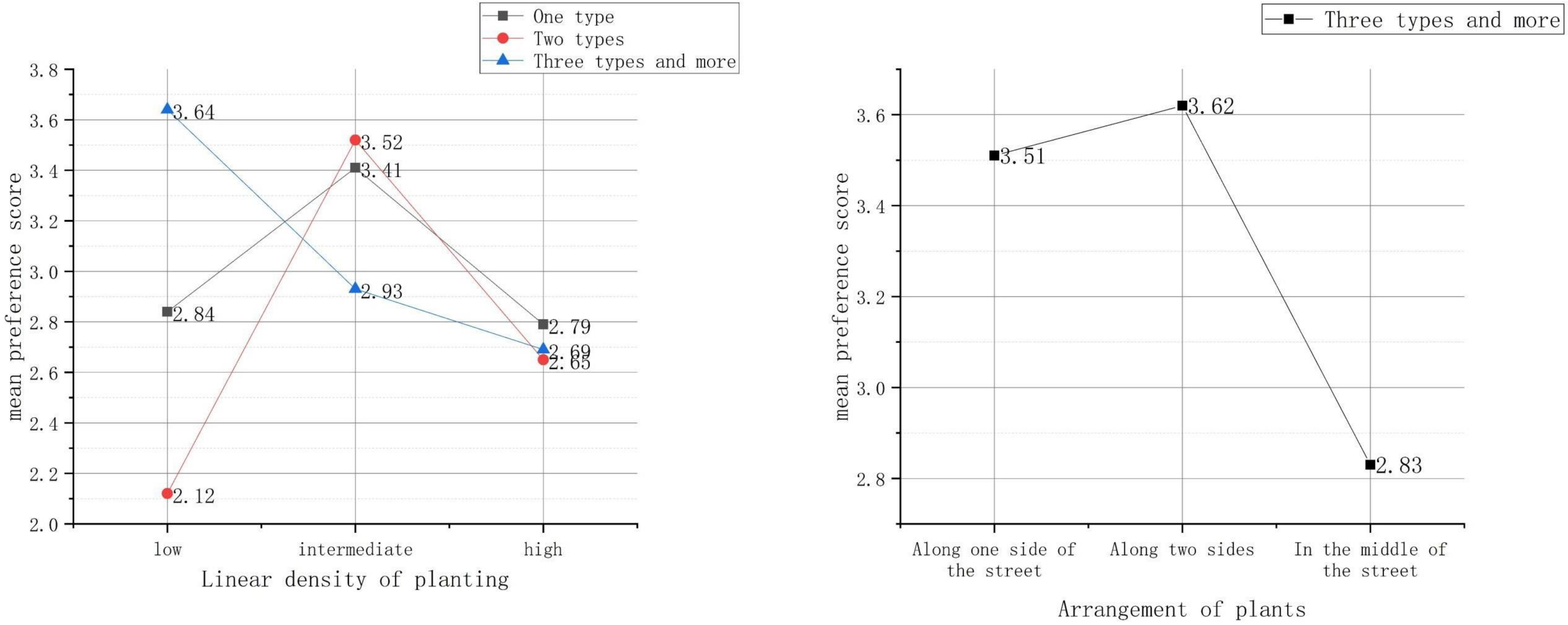

Fig 4 Linear density of planting and arrangement of plant

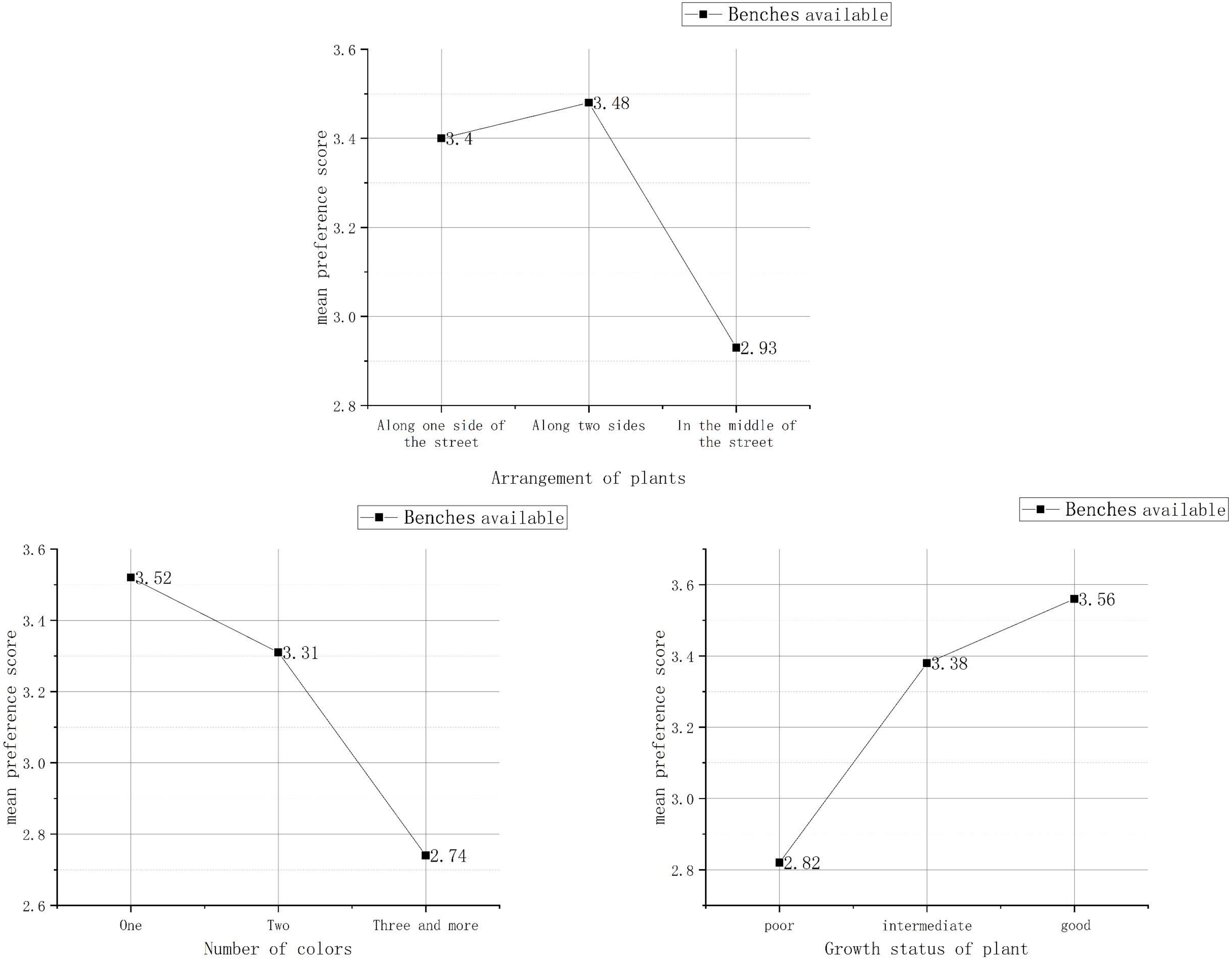

Fig 5 Preference evaluation under the influence of benches and plant factors I

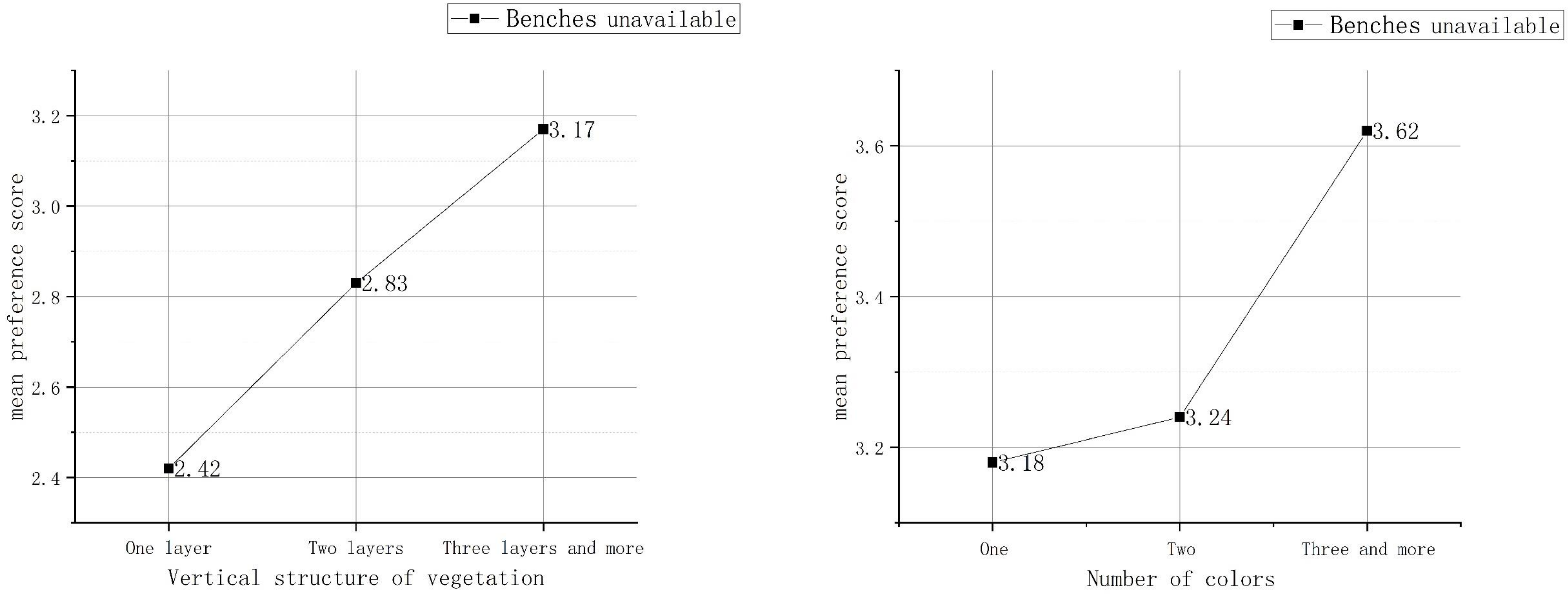

Fig 6 Preference evaluation under the influence of benches and plant factors II
